# Betaine Attenuates Monocrotaline-Induced Pulmonary Arterial Hypertension in Rats via Inhibiting Inflammatory Response

**DOI:** 10.3390/molecules23061274

**Published:** 2018-05-26

**Authors:** Jia-mei Yang, Ru Zhou, Min Zhang, Huan-ran Tan, Jian-qiang Yu

**Affiliations:** 1 Department of Pharmacology, Ningxia Medical University, Yinchuan 750004, China; m15809581390@163.com (J.Y.); zhou-ru926@163.com (R.Z.); 18209511903@163.com (M.Z.); 2 Ningxia Hui Medicine Modern Engineering Research Center and Collaborative Innovation Center, Ningxia Medical University, Yinchuan 750004, China; 3 Key Laboratory of Hui Ethnic Medicine Modernization, Ministry of Education, Ningxia Medical University, Yinchuan 750004, China; 4 Department of Pharmacology, Peking University, Health Science Center, Beijing 100191, China

**Keywords:** betaine, pulmonary arterial hypertension, inflammatory response

## Abstract

Background: Pulmonary arterial hypertension (PAH) is characterized by increased pulmonary vascular resistance, leading to right ventricular failure and death. Recent studies have suggested that chronic inflammatory processes are involved in the pathogenesis of PAH. Several studies have demonstrated that betaine possesses outstanding anti-inflammatory effects. However, whether betaine exerts protective effects on PAH by inhibiting inflammatory responses in the lungs needs to be explored. To test our hypothesis, we aimed to investigate the effects of betaine on monocrotaline-induced PAH in rats and attempted to further clarify the possible mechanisms. Methods: PAH was induced by monocrotaline (50 mg/kg) and oral administration of betaine (100, 200, and 400 mg/kg/day). The mean pulmonary arterial pressure, right ventricular systolic pressure, and right ventricle hypertrophy index were used to evaluate the development of PAH. Hematoxylin and eosin staining and Masson staining were performed to measure the extents of vascular remodeling and proliferation in fibrous tissue. Monocyte chemoattractant protein-1 (MCP-1) and endothelin-1 (ET-1) were also detected by immunohistochemical staining. Nuclear factor-κB (NF-κB), tumor necrosis factor alpha (TNF-α), and interleukin-1β (IL-1β) were assessed by Western blot. Results: This study showed that betaine improved the abnormalities in right ventricular systolic pressure, mean pulmonary arterial pressure, right ventricle hypertrophy index, and pulmonary arterial remodeling induced by monocrotaline compared with the PAH group. The levels of MCP-1 and ET-1 also decreased. Western blot indicated that the protein expression levels of NF-κB, TNF-α, and IL-1β significantly decreased (*p* < 0.01). Conclusion: Our study demonstrated that betaine attenuated PAH through its anti-inflammatory effects. Hence, the present data may offer novel targets and promising pharmacological perspectives for treating monocrotaline-induced PAH.

## 1. Introduction

Pulmonary arterial hypertension (PAH) is a refractory disease defined by a mean pulmonary artery pressure (mPAP) at or above 25 mmHg; it is characterized by elevated pulmonary vascular resistance and arterial pressures and driven by a progressive pulmonary vasculopathy that leads to right ventricular hypertrophy (RVH), right ventricular failure, and death [1,2].

The pathogenic mechanisms underlying PAH include vascular remodeling, inflammation, vasoconstriction, and thrombosis; PAH is generally assumed to involve an interaction of multiple factors [3]. In the past decade, an increasing number of studies have addressed the molecular pathway involved in the development of PAH [4]. Inflammation is considered an important contributor to vascular remodeling in PAH [5,6]. The excessive proliferation of pulmonary arterial smooth muscle cells and perivascular inflammation lead to PAH progression, but they are not specifically targeted by current therapies [7].

Nuclear factor-κB (NF-κB) is a central regulator of innate and adaptive immune responses. Its function is accomplished through the induction of genes, some of which promote inflammation, leukocyte migration, and activation [8]. The NF-κB and tumor necrosis factor alpha (TNF-α) signaling pathways have become hotspots for research, and they play a predominant role in the pathogenesis of PAH [9,10,11]. The primary importance of inflammation is illustrated by successful therapies using an IL-1 receptor antagonist and antibodies to monocyte chemotactic protein-1 in a monocrotaline (MCT)-induced PAH model [12]. Thus, the anti-inflammatory effects and interference of NF-κB may serve as a therapeutic target for PAH inhibition.

Known as “red gold” in northwest China, Chinese wolfberry (*Lycium barbarum*) is a valuable resource of traditional Chinese herbal medicine in NingXia and a health food in many countries worldwide. Modern pharmacological studies revealed that wolfberry exhibits various activities, such as antioxidant, anti-aging, neuroprotective, and anti-Alzheimer’s disease properties [13]. Betaine, a highly important alkaloid isolated from Chinese wolfberry, mainly exists in berries, leaves, and stems, and possesses various pharmacological activities, such as anti-inflammation, anti-fibrosis, and anti-oxidation [14,15,16]. Notably, a series of studies has found that betaine possesses outstanding anti-inflammatory abilities and exerts protective effects by suppressing the NF-κB signaling pathways; these activities include alleviating lipopolysaccharide-induced memory impairment, high-fat diet plus carbon tetrachloride-induced liver fibrosis, and isoproterenol-induced acute myocardial injury [17,18,19,20,21]. These results aroused our interest and suggested that betaine may be a potential drug for PAH.

In this regard, we hypothesized that betaine can exert protective effects on MCT-induced PAH in rats. This experiment was designed to investigate the protective abilities of betaine and then explore the anti-inflammatory effects of betaine on MCT-induced PAH.

## 2. Materials and Methods

### 2.1. Materials and Experimental Design

Healthy adult male Sprague-Dawley rats (age: 8–10 weeks; weights: 220 and 250 g) were obtained from the Experimental Animal Center of Ningxia Medical University (Ningxia, China). The rats were given feed and water ad libitum in a temperature-controlled environment with a 12 h/12 h light/dark cycle. The adopted animal experimental protocol was approved by the Animal Experimental Committee Ningxia Medical University. Betaine was purchased from Sigma-Aldrich (St. Louis, MO, USA) with HPLC purity of 98% and dissolved in 0.9% saline. MCT (Sigma-Aldrich) was dissolved in PBS and 0.1 N HCl. The pH was adjusted to 7.4, and the volume was raised to achieve a final concentration of 20 mg/mL. The MCT solution was sterilized by filtration through a 0.45 mL syringe filter, aliquoted, and stored at 20 °C. The rats were randomized into six groups (15 rats in each group) as follows: (1) control group, (2) MCT group, (3) betaine 100 mg/kg/day group, (4) betaine 200 mg/kg/day group, (5) betaine 400 mg/kg/day group, and (6) sildenafil (25 mg/kg/day) group. The PAH model was established by a single abdominal subcutaneous injection of 50 mg/kg MCT (control animals were subcutaneously injected abdominally with saline) at day 0. Subsequently, all rats in the control group and MCT group received the vehicle through intragastric administration daily from day 21 to day 42. Meanwhile, the rats in the other groups received betaine at three doses or sildenafil from day 21 to day 42 in the same manner.

### 2.2. Assessment of Hemodynamics

At 3 weeks after betaine administration, rats were anesthetized with urethane (100 mg/kg IP) for invasive hemodynamic measurements. The anesthetized rats were then intubated and mechanically ventilated with ambient air. A 2-cm-long incision was created to separate the blood vessels. A polyethylene catheter was inserted (Beijing Union Medical College, Department of Pathophysiology, Beijing, China) and connected with pressure transducers (Alcott Biotech, Shanghai, China) into the right external jugular vein, through the right atrium and right ventricle, and eventually to the pulmonary artery to obtain hemodynamic measurements. After hemodynamic assessment, the rats were sacrificed by cervical dislocation under anesthesia. The abdominal cavity was opened, the diaphragm was incised to expose the pleural cavity, the ribs were cut away to access the lungs and heart, and the whole lung and heart were excised. The left lung tissues removed from all the experimental groups were fixed in 4% paraformaldehyde and embedded in paraffin for histologic evaluation (hematoxylin and eosin (H&E) staining, Masson staining, and immunohistochemical analysis), and the right lungs were frozen in liquid nitrogen for Western blot analysis.

### 2.3. Measurement of RVHI

At the end of the hemodynamic measurements, the hearts were quickly dissected and weighed to determine RVH. The left ventricle (LV) wall, interventricular septum (S), and right ventricle (RV) wall were weighed separately. The index of RVH was expressed as the weight ratio of RV to LV plus the septum (RV/(LV + S)). After weight measurement, the RVs from all the experimental groups were fixed in 4% paraformaldehyde and embedded in paraffin for morphometric analysis.

### 2.4. H&E and Masson Staining

After fixation in 4% paraformaldehyde for 48 h, the lung tissue was embedded in paraffin and cut into 5 μm tissue sections. H&E and Masson staining of paraffin-embedded sections of lung tissues were performed to evaluate pathologic changes and collagen fiber hyperplasia under light microscopy. The ratio of vascular wall thickness and the ratio of the vascular wall area in the middle and small arterioles with an outer diameter of 50–150 μm were measured by using an image analyzing system (Image-Pro Plus, Media Cybernetics, Silver Spring, MD, USA). Simultaneously, two indices reflecting the vessel thickness were calculated: the ratio of vascular wall thickness (WT%) = 100% × (outer diameter of the pulmonary arterioles − inner diameter of the pulmonary arterioles)/(outer diameter of the pulmonary arterioles); the ratio of vascular wall area (WA%) = 100% × (transection area of the walls of pulmonary arterioles)/(cross-sectional area of pulmonary arterioles). For each animal, over 10 randomly chosen vessels that were nearly round or oval in shape were measured and averaged by an observer blinded to the experimental groups.

### 2.5. Evaluation of RV Remodeling

RV tissue was preserved in 4% paraformaldehyde for 48 h, and H&E staining of paraffin-embedded sections of RV tissue was performed. From the transverse sections of paraffin-embedded RV walls, the cross-sectional area per cardiomyocyte was determined using the aforementioned image analyzing system. The mean values were obtained to determine the average diameter (AD) of myocardial cells and myocardial nuclear density (MND) for the evaluation of RV remodeling.

### 2.6. Immunohistologic Study

Additional lung sections were immunostained to identify the location of the protein expression of monocyte chemoattractant protein-1 (MCP-1) and endothelin-1 (ET-1) in the lungs. After rehydration and antigen retrieval, endogenous peroxidase activity was quenched with 0.3% hydrogen peroxide, and non-specific binding was blocked with 3% BSA for 1 h at room temperature. Subsequently, the sections were incubated for 2 h with primary antibodies for MCP-1 (1:200) and ET-1 (1:1000) (Abcam, Cambridge, United Kingdom), followed by incubation with corresponding secondary antibodies (Zhongshan-Golden Bridge Biological Technology, Beijing, China). The sections were then visualized with 3-3ʹ diaminobenzidine hydrochloride substrate (1:3000; Applygen technology, Beijing, China) and counterstained by hematoxylin.

### 2.7. Western Blot

Protein was extracted from lung tissues frozen in liquid nitrogen in lysis buffer using protein extraction reagent (BCA Protein Quantitative Kit, Kaiji, Nanjing, China). Lysates were centrifuged (2 × 10^4^ rpm) at 4 °C for 10 min. Protein expression (40 μg/lane) was detected by SDS PAGE (8–15% gel), and proteins were electroblotted onto nitrocellulose membranes and then incubated by blocking with PBST containing 5% skim milk blocking buffer at room temperature for 2 h. Subsequently, the immunoblots were washed and probed with rabbit monoclonal anti-NF-κB antibody (1:500), rabbit polyclonal anti-TNF-α antibody (1:500), rabbit polyclonal anti-interleukin-1β (anti-IL-1β) antibody (1:300), and polyclonal anti-β-actin antibody (1:2000) at 4 °C for 48 h. The membranes were washed and incubated with goat anti-rabbit IgG (1:2000) (Zhongshan-Golden Bridge Biological Technology, Beijing, China) as secondary antibody. The protein bands were visualized with enhanced chemiluminescence (ECL) reagents (Applygen technology, Beijing, China) and quantified by lumino-analyzer (Advansta, Menlo Park, CA, USA). The contents of NF-κB, TNF-α, and interleukin-1β (IL-1β) proteins were analyzed by densitometric quantification using Bio-Rad Quantity One software (Bio-Rad Company, Hercules, CA, USA).

### 2.8. Data Analysis

Results were presented as mean ± standard error of the mean (SEM) and analyzed with SPSS 18.0. All data were analyzed by one-way ANOVA and Newman-Keuls-Student test for multiple comparisons. The experiment was unpaired design, and the hypothesis testing was two-tailed test. For all statistical tests, statistical significance was set at *p* ≤ 0.05 and *p* ≤ 0.01.

## 3. Results

### 3.1. Betaine Inhibits the Development of PAH and RV Hypertrophy

**Figure 1:**
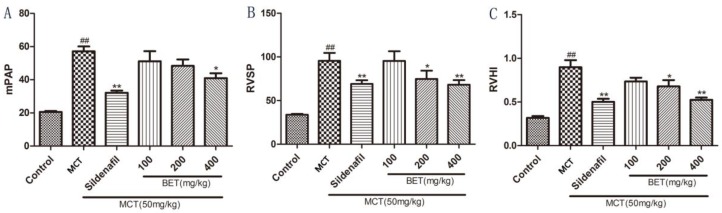
Effects of betaine on monocrotaline-induced pulmonary hypertension. (**A**) Effect of betaine (BET) on the mean pulmonary arterial pressure (mPAP). (**B**) Effect of BET on the right ventricular systolic pressure (RVSP). (**C**) Effect of BET on the right ventricular hypertrophy index (RVHI). Data are expressed as mean ± standard error of the mean (*n* = 6). ^##^ *p* < 0.01 vs. control group, * *p* < 0.05, ** *p* < 0.01 vs. MCT group. MCT: monocrotaline-induced group.

We evaluated the effects of betaine on MCT-induced lung and heart injury by detecting the mPAP, right ventricular systolic pressure, and RVH index (RVHI = RV/(LV + S)). Rats injected with MCT consistently developed significant PAH within 21 days compared with the control animals (*p* < 0.01). The rats treated with betaine (400 mg/kg) demonstrated a significantly lower mPAP than the MCT animals (*p* < 0.05; Figure 1A). In the MCT group, obvious RV systolic pressure (RVSP) elevation developed as a consequence of increased pulmonary arterial pressures. Betaine treatment reduced the RVSP (*p* < 0.05 and *p* < 0.01) in a manner similar to sildenafil treatment (*p* < 0.01; Figure 1B). Betaine treatment also decreased the RVHI (*p* < 0.05 and *p* < 0.01) similarly to sildenafil treatment (*p* < 0.01; Figure 1C). These results indicated that betaine treatment could protect the lungs and heart in rats with MCT-induced PAH.

### 3.2. Betaine Reverses Pulmonary Vascular and RV Remodeling and Inhibits Inflammatory Cells Infiltration

**Figure 2:**
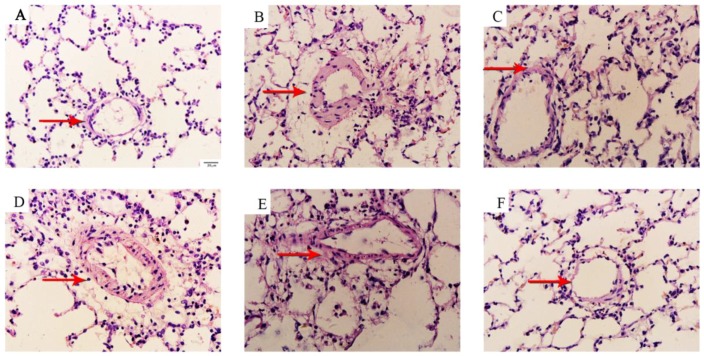
(**A**–**F**) Effects of betaine on medial wall thickness and small pulmonary arteries assessed by HE staining (magnification ×400). (**A**) Control group, (**B**) MCT group, (**C**) sildenafil group, (**D**) betaine 100 mg/kg group, (**E**) betaine 200 mg/kg group, and (**F**) betaine 400 mg/kg group.

**Figure 3:**
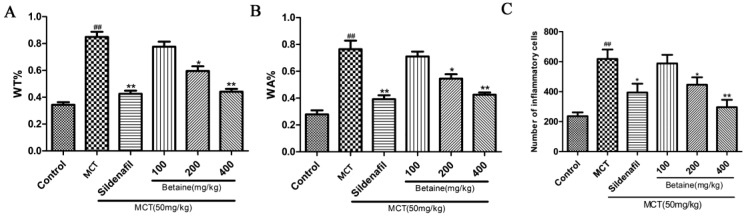
Effects of betaine on (**A**) the ratio of vascular wall thickness, (**B**) the ratio of vascular wall area and (**C**) number of inflammatory cells. Data are expressed as mean ± standard error of the mean (*n* = 6). ^##^ *p* < 0.01 vs. control group, * *p* < 0.05, ** *p* < 0.01 vs. MCT group. MCT: MCT-induced group, WT%: ratio of vascular wall thickness = 100% × (outer diameter of the pulmonary arterioles∔inner diameter of the pulmonary arterioles)/(outer diameter of the pulmonary arterioles); WA%: ratio of vascular wall area = 100% × (transection area of the walls of pulmonary arterioles)/(cross-sectional area of pulmonary arterioles).

We performed H&E and Masson staining to study the pathologic changes in the middle and small arteries (>50 to <200 μm) of the lungs. The arteries of the lungs in the control group were characterized by a thin medial wall and large lumen (Figure 2A); the medial wall thickness and vascular stenosis of the small pulmonary arteries significantly increased in the MCT-treated rats than in the control (Figure 2B). The betaine-treated rats demonstrated a significant improvement in vascular morphology (Figure 2F) that was similar to the sildenafil-treated rats (Figure 2C). Accordingly, the WT% (Figure 3A) and WA% (Figure 3B) of pulmonary arterioles and number of inflammatory cells around the vessel wall and tissues (Figure 3C) remarkably increased in the rats treated with MCT (*p* < 0.05 and *p* < 0.01) than in the control. Sildenafil and betaine (200 and 400 mg/kg) successfully reversed the MCT-induced increases in WT% and WA% of the pulmonary arterioles and number of inflammatory cells around the vessel wall and tissues (*p* < 0.05 and *p* < 0.01).

**Figure 4:**
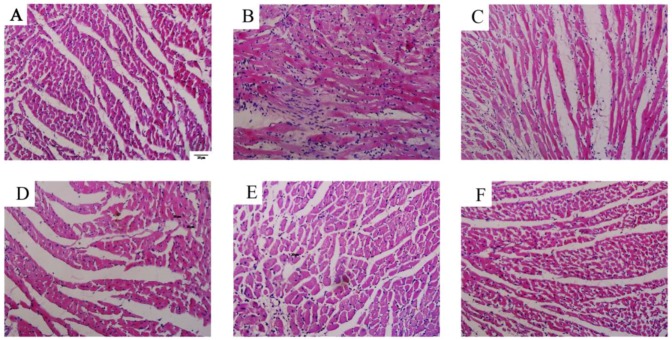
(**A**–**F**) Effects of betaine on myocardial cells assessed by HE staining (magnification ×200). (**A**) Control group, (**B**) MCT group, (**C**) sildenafil group, (**D**) betaine 100 mg/kg group, (**E**) betaine 200 mg/kg group, and (**F**) betaine 400 mg/kg group.

**Figure 5:**
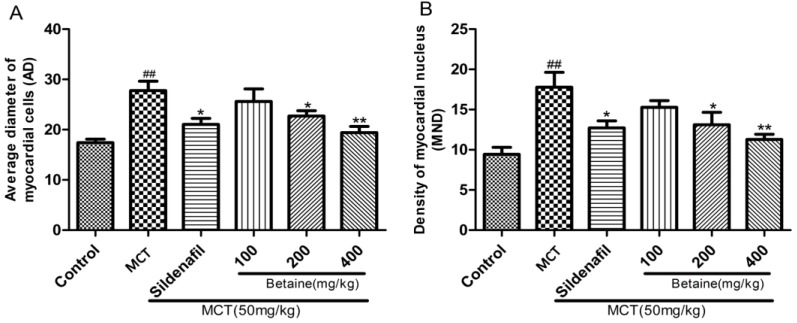
(**A)** Effects of betaine on right ventricle remodeling and (**B)** Average diameter (AD) of myocardial cells and myocardial nuclear density (MND). Data are shown as mean ± standard error of the mean (*n* = 6). ^##^ *p* < 0.01 vs. control group, * *p* < 0.05, ** *p* < 0.01 vs. MCT group. MCT: MCT-induced group.

H&E staining of RV tissues demonstrated that the myocardial cells in the control group showed good continuity, regular arrangements, and small gaps (Figure 4A). Meanwhile, the myocardial cells of the MCT-injected rats demonstrated significant hyperplasia and hypertrophy, disordered arrangement, sparseness, swollen cytoplasm, and increased myocardial cell gap and area of myocardial fibrosis hyperplasia relative to those in the control (Figure 4B). The betaine-treated rats exhibited significant improvements in the abovementioned aspects (Figure 4F) similar to the sildenafil-treated rats (Figure 4C). Accordingly, the AD of the myocardial cells (Figure 5A) and MND (Figure 5B) of the MCT-treated rats significantly increased relative to those of the control rats (*p* < 0.01). Betaine treatment decreased the AD and MND to a similar extent as in sildenafil treatment compared with the MCT rats (*p* < 0.01).

**Figure 6:**
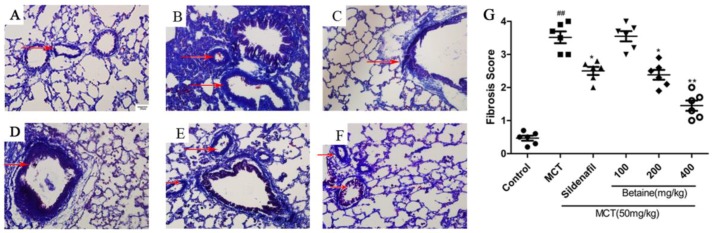
(**A**–**G**) Effect of betaine on collagen fiber hyperplasia assessed by Masson staining (magnification ×400). (**A**) Control group, (**B**) MCT group, (**C**) sildenafil group, (**D**) betaine 100 mg/kg group, (**E**) betaine 200 mg/kg group, (**F**) betaine 400 mg/kg group and (**G**) the grade of fibrosis with Masson staining. 0, no fibrosis; 1+, fibrosis present; 2+, mild fibrosis; 3+, moderate fibrosis; 4+, severe fibrosis. ^##^ *p* < 0.01 vs control group; * *p* < 0.05, ** *p* < 0.01 vs. MCT group.

Masson staining showed few collagen fibers and inflammatory cells in the field of vision in the control group (Figure 6A), but the MCT group displayed numerous disorganized, proliferating collagen fibers within the vessel wall and surrounding tissue, as well as many inflammatory cells (Figure 6B). The betaine-treated rats showed significant improvements in collagen fiber proliferation and inflammatory cell infiltration (Figure 6F), similar to the sildenafil-treated rats (Figure 6C). These histological results also indicate that betaine could inhibit fibrosis and exert protective effects on the lungs and heart in rats with MCT-induced PAH.

### 3.3. Immunohistochemical Analyses Revealed Betaine Attenuated MCP-1, ET-1 Expression in Lung

**Figure 7:**
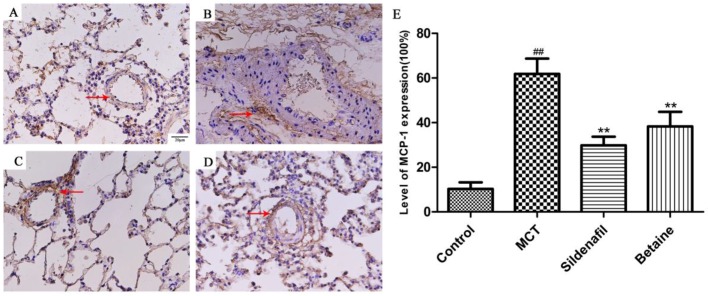
(**A**–**D**) Effects of betaine on the expression of MCP-1 in lungs (magnification ×400). Data are shown as mean ± standard error of the mean (*n* = 6). ^##^ *p* < 0.01 vs control group; ** *p* < 0.01 vs MCT group. (**A**) Control group, (**B**) MCT group, (**C**) betaine 400 mg/kg group, (**D**) The bar graph reflects level of MCP-1 expression.

**Figure 8:**
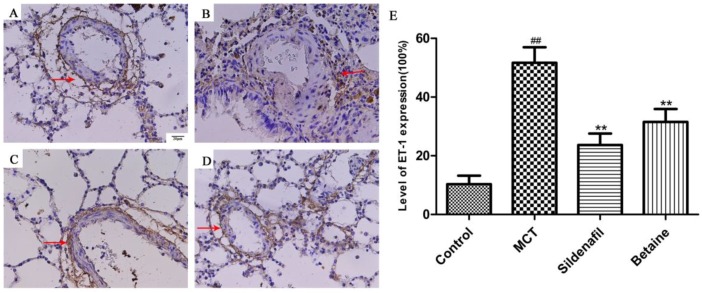
(**A**–**D**) Effects of betaine on the expression of ET-1 in lungs (magnification ×400). Data are shown as mean ± standard error of the mean (*n* = 6). ^##^ *p* < 0.01 vs control group; ** *p* < 0.01 vs MCT group. (**A**) Control group, (**B**) MCT group, (**C**) betaine 400 mg/kg group, (**D**) The bar graph reflects level of ET-1 expression.

The expression levels of MCP-1 of ET-1 reflect the severity of the inflammatory response in the lungs and were determined through immunohistochemical staining of lung tissue slices. The expression levels of MCP-1 and ET-1 in the lungs markedly increased in the MCT group (Figure 7B and Figure 8B) than in the control group (Figure 7A and Figure 8A), but this change was significantly lessened by betaine (400 mg/kg; Figure 7D and Figure 8D) and sildenafil (Figure 7C and Figure 8C). The results suggest that betaine treatment inhibited the MCT-induced expression of MCP-1 and ET-1.

### 3.4. Betaine Attenuates NF-κB, TNF-α, and IL-1β Protein Expression

**Figure 9:**
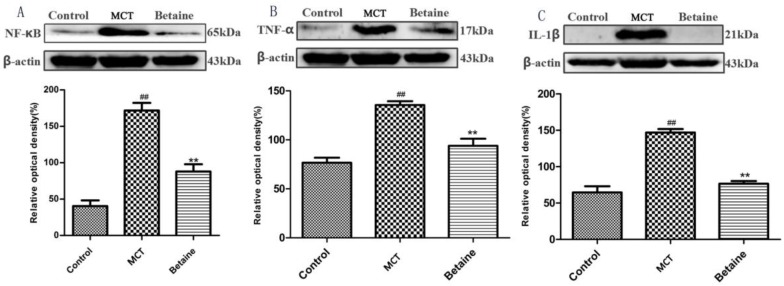
(**A**–**C**) Effects of betaine on the protein expression of nuclear factor-κB (NF-κB), tumor necrosis factor alpha (TNF-α) and interleukin-1β (IL-1β) assessed by Western blot. Representative western blot band and expression of protein level of (**A**) NF-κB, (**B**) TNF-α and (**C**) IL-1β; β-actin was used for normalization. Data are shown as mean ± standard error of the mean (*n* = 6). ^##^ *p* < 0.01 vs control group; ** *p* < 0.01 vs MCT group.

To prove that betaine plays a role in inhibiting the inflammatory response in MCT-induced PAH, we further tested the protein expression of NF-κB, TNF-α, and IL-1β, which involved the inflammatory response. Our Western blot analysis results demonstrated that the expression levels of NF-κB, TNF-α, and IL-1β were up-regulated in the MCT group than in the control group (*p* < 0.01, Figure 9A–C, respectively), and betaine treatment (400 mg/kg) significantly reduced the protein expression of NF-κB, TNF-α, and IL-1β (*p* < 0.01, Figure 9A–C, respectively).

## 4. Discussion

PAH is a debilitating and life-threatening disease with an estimated prevalence of 52 cases per million. Moreover, the prevalence of PAH is about 1% of the general population, which increases to 10% of individuals aged over 65 years [22]. The complex cellular and molecular pathology changes contribute to the remodeling of the pulmonary artery and small vasculature in the lungs, ultimately leading to increased pulmonary vascular resistance, right heart failure, and death [23]. Over the past 20 years, substantial efforts have been made to develop effective therapies for patients with PAH. Current therapies such as prostanoids, endothelin receptor antagonists, or phosphodiesterase type 5 inhibitors largely impact vasoconstriction pathology, which reduces the symptoms of disease by promoting vasodilation [24]. These therapies do not reduce disease progression or produce regression, and they only have a limited impact on mortality. Despite current therapies, most patients still die from the disease or fail to respond adequately to medical therapy, presenting a 5-year survival of 59% [25]. Given that conventional pulmonary vasodilators have limited efficacy for the treatment of severe PAH, novel drugs are required urgently to exert better therapeutic effects on PAH.

Betaine, one of the most important alkaloids isolated from Chinese wolfberry, possesses beneficial properties against inflammation, fibrosis, and oxidation [18,26,27]. This study is the first to demonstrate that intragastric administration of betaine exerted protective effects on MCT-induced PAH in rats. In the present study, we explored that betaine treatment alleviated the damage of the lungs and heart and significantly decreased mPAP, RVSP, and RVHI. Histology verification demonstrated that WT% and WA% of the pulmonary vasculature in the smooth muscle layer of pulmonary arterioles and AD and MND of myocardial cells improved, whereas hyperplasia of fibrous tissues in the lungs decreased. Meanwhile, the expression levels of MCP-1 and ET-1 in the lungs decreased. Western blot results revealed that betaine treatment also down-regulated the protein expression of NF-κB, TNF-α, and IL-1β significantly. These findings suggest that betaine could attenuate vascular remodeling by regulating the NF-κB signaling pathway, thereby inhibiting the inflammatory response.

Animal models have been used to study PAH pathogenesis and the effects of drug interventions [28,29]. Monocrotaline is a pyrrolizidine alkaloid present in the stems, leaves, and seeds of the plant Crotalaria spectabilis and in all the other plants of the Crotalaria genus. MCT-induced PAH is similar to human PAH in terms of hemodynamic and histopathological severity, including upregulation of inflammatory cytokines, vascular remodeling, and proliferation of smooth muscle cells, endothelial dysfunction, and RV failure [30,31]. Furthermore, proinflammatory cytokines, such as IL-1, IL-6, and TNF-α, are excessively produced in animals treated with MCT [32]. Previous studies have examined the efficacy of cytokine antagonists, especially IL-1 and IL-6 blockage in the MCT model [33].

The role of inflammation is increasingly being recognized in the pathogenesis of PAH [34]. Recent studies have described inflammation as a characteristic feature of many forms of PAH in both humans and animals [35]. Levels of proinflammatory cytokines and chemokines are increased in lung tissues and blood of patients with PAH. In addition, the effect of inflammation in PAH development was further confirmed by the clinical improvements observed in patients after steroid treatment or immune suppressor administration.

NF-kB is known as a central regulator of targeted genes associated with inflammation, dysfunction of endothelial cells, and angiogenesis [8]. Activation of the classical NF-kB pathway induces the expression of various genes encoding proinflammatory cytokines and chemokines, such as IL-1β and IL-6; therefore, NF-kB can promote vascular inflammation by chemotaxis of leukocytes [36,37]. Some studies showed that the role of NF-kB in PAH is of great importance: pulmonary artery smooth muscle cells and macrophages derived from patients with idiopathic PAH show increased NF-kB activation, and NF-kB inhibition ameliorates MCT-induced PAH [38,39].

NF-κB is recognized as a transcription factor that mediates the expression of TNF-α, particularly in endothelial vascular cells. TNF-α is a proinflammatory cytokine with potent modulatory effects on pulmonary circulation [34]. Some researchers have reported that the TNF-α-mediated inhibition of the metabolic enzyme pyruvate dehydrogenase contributes to the pathogenesis of PAH, and elevated serum TNF-α levels are observed in patients with pulmonary hypertension secondary to chronic thromboembolic disease and connective tissue disease [40]. In animal studies, TNF-α was shown to increase pulmonary vascular reactivity, reduce prostacyclin production in pulmonary artery smooth muscle cells, and potentiate platelet-activating factor-induced pulmonary vasoconstriction [41]. Overexpression of TNF-α results in severe PAH and emphysema in mice. Moreover, suppression of TNF production by high doses of pentoxyfylline reduces both systemic and pulmonary vascular resistance. Collectively, these data provide strong experimental evidence that TNF-α plays an important role in pulmonary vascular physiology [42].

We also focused on the inflammatory cytokine MCP-1 because of its important role in patients with idiopathic PAH and MCT-induced PAH [43]. The expression of MCP-1 increases in several types of PAHs, and the level of MCP-1 in circulation is correlated with disease severity [44]. Furthermore, NF-kB regulates MCP-1 in vascular smooth muscle cells [45].

The endothelium plays an important role in the regulation of vascular function by producing a large number of biologically active substances that participate in the regulation of vascular tone, cell growth, inflammation, and thrombosis [46,47]. Endothelin (ET)-1 is a potent vasoconstrictor peptide originally isolated from endothelial cells [48,49]. Its production is stimulated in a variety of different cell types under the influence of risk factors for cardiovascular disease and during the development of cardiovascular disease [50]. The expression levels of MCP-1 and ET-1 reflect the severity of the inflammatory response in the lungs, and betaine treatment attenuated the expression of MCP-1 and ET-1 induced by MCT.

Pulmonary fibrosis is often complicated by PAH, and it is implicated as a major participant in PAH and is currently being studied as a new therapeutic target [51,52]. The development of MCT-induced pulmonary fibrosis is characterized by an initial inflammatory process with upregulation of pro-fibrotic markers; it increases progressively in a time-dependent manner. IL-1β, which is associated with PAH, may also contribute to the pathogenesis of pulmonary fibrosis. TNF-𝛼, which stimulates fibroblasts and facilitates collagen production, has been demonstrated to be elevated in the lungs of patients with idiopathic pulmonary fibrosis [53,54]. IL-1β-induced fibrosis is associated with an increase in TNF-α expression, suggesting a mechanistic association [55,56]. Our Masson staining experimental results proved that betaine-treated rats demonstrated a significant improvement in the proliferation of collagen fibers and inflammatory cell infiltration; these effects were similar to the results observed in sildenafil-treated rats. Western blot analysis demonstrated that the expression level of IL-1β was up-regulated in the MCT group compared with the control group, and treatment with betaine (400 mg/kg) significantly reduced the protein expression of IL-1β. 

Sildenafil, a phosphodiesterase type 5 inhibitor, is recognized as an effective treatment for PAH [57,58]. Therefore, as the control principle of the experimental design, we chose sildenafil as a positive drug group in this study [59]. In the current study, sildenafil exerted a protective effect on MCT-induced PAH as previously described [60]. In addition, our results proved the validity of our experimental design and reliability of the protective effects of betaine on PAH. Firstly, betaine, which has the advantages of multi-targeted efficacy, low toxicity and low cost, shows promise in recent research. Secondly, expensive medical expenses are also the main causes of patients’ oppositional and unmotivated mental behavior in clinical treatment. Limited therapeutic scheduling for PAH can be attributed to insufficient drugs to some extent. Betaine might be a potential drug for PAH and additional investigations are required to ensure that the concrete molecular targets mediate the protective effects of betaine on PAH.

## 5. Conclusions

Our novel findings suggested that betaine treatment reduced mPAP and RVSP, alleviated pulmonary arteries and right ventricle remodeling, and attenuated fibroplasia in the lungs of rats with MCT-induced PAH. The mechanism was through inhibiting the inflammatory reaction and down-regulating the NF-κB signaling pathway. Further studies are warranted before betaine can be applied to the clinical treatment of PAH.
